# Statins inhibit paclitaxel-induced PD-L1 expression and increase CD8^+^ T cytotoxicity for better prognosis in breast cancer

**DOI:** 10.1097/JS9.0000000000001582

**Published:** 2024-05-13

**Authors:** Lei Li, Hongbin Wang, Shiyuan Zhang, Song Gao, Xiuxin Lu, You Pan, Wei Tang, Rong Huang, Kun Qiao, Shipeng Ning

**Affiliations:** a Department of Breast Surgery, The Second Affiliated Hospital of Guangxi Medical University, Nanning; b Department of Breast Surgery, Harbin Medical University Cancer Hospital, Harbin, People’s Republic of China; c Department of Pathology, University of Otago, Dunedin, New Zealand

**Keywords:** breast cancer, combination therapy, paclitaxel, PD-L1, statins

## Abstract

**Background:**

In recent years, the widespread use of lipid-lowering drugs, especially statins, has attracted people’s attention. Statin use may be potentially associated with a reduced risk of breast cancer.

**Objective:**

To explore the relationship between statin use and cancer risk. And further explore the potential role of statins in the adjuvant treatment of breast cancer.

**Methods:**

Data for the Mendelian randomization portion of the study were obtained from genome-wide association studies of common cancers in the UK Biobank and FinnGen studies and from the Global Lipid Genetics Consortium’s low density lipoprotein (LDL). In addition, the impacts of statins and chemotherapy drugs on breast cancer were examined using both in *vitro* and in *vivo* models, with particular attention to the expression levels of the immune checkpoint protein PD-L1 and its potential to suppress tumor growth.

**Results:**

Data from about 3.8 million cancer patients and ~1.3 million LDL-measuring individuals were analyzed. Genetically proxied HMGCR inhibition (statins) was associated with breast cancer risk reduction (*P*=0.0005). In *vitro* experiments showed that lovastatin significantly inhibited paclitaxel-induced PD-L1 expression and assisted paclitaxel in suppressing tumor cell growth. Furthermore, the combination therapy involving lovastatin and paclitaxel amplified CD8^+^ T-cell infiltration, bolstering their tumor-killing capacity and enhancing in *vivo* efficacy.

**Conclusion:**

The utilization of statins is correlated with improved prognoses for breast cancer patients and may play a role in facilitating the transition from cold to hot tumors. Combination therapy with lovastatin and paclitaxel enhances CD8^+^ T-cell activity and leads to better prognostic characteristics.

## Introduction

Highlights

- Statin use associated with reduced breast cancer risk.
- Lovastatin inhibits paclitaxel-induced PD-L1 expression.
- Combination of lovastatin and paclitaxel enhances CD8^+^ T cell cytotoxicity.
- Lovastatin and paclitaxel combination treatment leads to better prognostic characteristics in mice.

Cancer stands as a prominent contributor to global mortality^1^. In 2018, the United States witnessed 609 640 deaths due to cancer, with 1 735 350 newly diagnosed cases^2^. Immunotherapy utilizing programmed death 1/programmed death ligand 1 (PD-1/PD-L1) inhibitors has gained approval for clinical use due to its notable benefits in augmenting cancer patient prognosis^3^. Nonetheless, a significant proportion of patients fail to achieve satisfactory responses to immunotherapy^4^. PD-1 in conjunction with PD-L1 serves to inhibit T-cell activation. Tumor cells exhibit heightened expression of PD-L1, which binds to PD-1 on T cells, resulting in immune evasion^5^.

The utilization of lipid-lowering agents, notably statins, has garnered substantial attention in recent years, primarily owing to their potential influence on cancer risks and clinical outcomes^6–9^. Originally designed for their cholesterol-reducing properties, these pharmaceuticals have revealed broader effects. Nielsen *et al*.^10^ undertook a retrospective analysis, wherein they compared the prognostic profiles of 18 721 patients who regularly administered statins with those of 277 204 patients who had never employed these medications. Their findings demonstrated that individuals utilizing statins exhibited reduced cancer-related mortality across a spectrum of 13 different cancer types, encompassing prominent malignancies such as prostate, breast, and pancreatic cancers^10^. Additionally, it is noteworthy that statins have been found to decrease PD-L1 expression in lung cancer and melanoma cell lines^11–13^. Enhanced anticancer effects of statins in combination with chemotherapy and targeted therapies have been observed in some cancer patients^14,15^. Therefore, statins may represent viable candidates for inclusion in adjuvant chemotherapy regimens.

**Figure 1:**
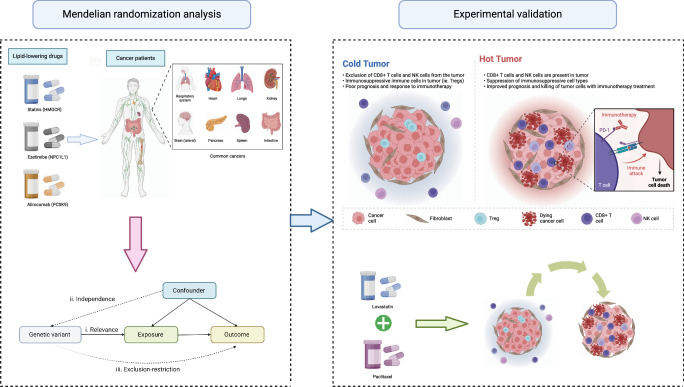
Flowchart of this study. This study first systematically explored the potential risk relationship between the use of lipid-lowering drugs and cancer patients based on MR methods. Then based on the MR results, we further studied the effects of statins on breast cancer in vivo and in vitro. Our results show that the combined use of lovastatin and paclitaxel helps improve the infiltration and tumor killing ability of CD8+ T cells and contributes to the transformation of ‘cold’ tumors into ‘hot’ tumors. ‘Cold’ tumors: these are tumors that are not recognized or attacked effectively by the immune system. They typically have low levels of immune cell infiltration and lack activation of immune responses. ‘Hot’ tumors: conversely, hot tumors are those that elicit a strong immune response. They typically have high levels of immune cell infiltration and exhibit signs of active immune recognition and response against the tumor cells.

Mendelian randomization (MR) can be conceptualized as a quasi-random natural experiment, offering enhanced resilience against biases inherent in traditional epidemiological designs, particularly when pleiotropic effects are absent^16,17^. In this study, we used 2-sample MR to investigate the association between the risk of 13 common cancers and 3 gene-agent lipid-lowering drugs: 3-hydroxy-3-methylglutaryl–CoA reductase (HMGCR) inhibitors (i.e. statins), Niemann-Pick C1–like 1 (NPC1L1) inhibitors (i.e. ezetimibe), and PCSK9 inhibitors (e.g. alirocumab). In addition, we established cellular models to further examine the MR results and to explore the role of lipid-lowering drugs in cancer cells. Results from further in *vivo* studies indicate that statins help to assist chemotherapy and enhance the tumor-killing ability of T cells. The flowchart of this study is shown in Figure 1.

## Methods

### Genetic associations for 13 cancer types

To provide for reproducibility of the study, a list of key resources for this study is presented in Table 1.

**Table 1:** Key resources table.

Reagent or resource	Source	Identifier
Antibodies		
PD-L1 (E1L3N) XP Rabbit Antibody	Cell Signaling Technology	Cat# 13684 RRID:AB_2687655
Rabbit Anti-GAPDH Monoclonal Antibody	Cell Signaling Technology	Cat# 2118 RRID:AB_561053
PD-L1 Polyclonal Antibody	Thermo Fisher Scientific	Cat# PA5-20343 RRID:AB_11153819
CD3 Monoclonal Antibody	Thermo Fisher Scientific	Cat# 16-0037-81 RRID:AB_468854
CD3 Monoclonal Antibody	Thermo Fisher Scientific	Cat# 16-0032-82 RRID:AB_468851
CD28 Monoclonal Antibody	Thermo Fisher Scientific	Cat# 16-0289-81 RRID:AB_468926
CD28 Monoclonal Antibody	Thermo Fisher Scientific	Cat# 16-0281-82 RRID:AB_46892
Chemicals, peptides, and recombinant proteins		
PBS	Gibco	Cat# 20012068
eBioscience Flow Cytometry Staining Buffer	Thermo Fischer Scientific	Cat#00-4222-26
Pierce Protease Inhibitor Mini Tablets	Thermo Fischer Scientific	Cat#A32953
Pierce Phosphatase Inhibitor Mini Tablets	Thermo Fischer Scientific	Cat#A32957
Pierce ECL Western Blotting Substrate	Thermo Fischer Scientific	Cat#32106
Bovine serum albumin (BSA)	Sigma	Cat#A2153-50G
Criterion XT Bis-Tris Precast Gels	Biorad	Cat#3450124
Cell Staining Buffer	Biolegend	Cat#420201
Western Blot Stripping Buffer	abcam	Cat#ab270550
Human IL-2 Recombinant Protein	Thermo Fischer Scientific	Cat#PHC0026
Paclitaxel	Sigma	Cat#Y0000698
Lovastatin	Sigma	Cat#L0790000
Critical commercial assays		
Purelink RNA Mini Kit	Thermo Fischer Scientific	Cat#12183025
QuantiTect Reverse Transcription kit	Qiagen	Cat#205313
WST-1 Assay Reagent	Abcam	Cat#ab155902
FAM FLICA™ Caspase-3/7 Kit	Biorad	Cat#ICT093
Other		
A lovastatin-elicited genetic program inhibits M2 macrophage polarization and enhances T cell infiltration into spontaneous mouse mammary tumors	Mira et al.	GEO: GSE42787
Large-scale association analysis identifies 13 new susceptibility loci for coronary artery disease	Schunkert *et al*.	N/A
Genetic association data summarizing breast cancer risk	Breast Cancer Association Consortium	N/A
Genetic associations of Brain glioblastoma and Malignant neoplasm	FinnGen study	N/A
Data for various cancers (Genetic associations)	UK Biobank	N/A
Genetic associations with LDL	Global Lipids Genetics Consortium	N/A
Experimental models: cell lines		
MCF-7	Cell Resource Center of Peking Union Medical College	N/A
MDAMB-231	Cell Resource Center of Peking Union Medical College	N/A
EMT6	Nanjing Cobioer Biosciences Co	Cat#CBP61111
Experimental models: Organisms/strains		
BALB/c mice	SPF Beijing Biotechnology Co	N/A
Oligonucleotides		
PD-L1 Forward	IDT	TGCCGACTACAAGCGAATTACTG
PD-L1 Reverse	IDT	CTGCTTGTCCAGATGACTTCGG
GAPDH Forward	IDT	CAAGATCATCACCAATGCCT
GAPDH Reverse	IDT	CCCATCACGCCACAGTTTCC
Software and algorithms		
GraphPad Prism 8	GraphPad Software	https://www.graphpad.com/
FlowJo	BD Biosciences	https://flowjo.com
Image Lab 6.1	Biorad, Image Lab	https://www.bio-rad.com/en-be/product/image-lab-software?ID=KRE6P5E8Z#fragment-6
Biorender	Biorender	https://biorender.com/
R v4.3.1	R Foundation	https://www.r-project.org/
R Studio	Posit	https://posit.co/download/rstudio-desktop/

Genetic association data summarizing breast cancer risk were sourced from the Breast Cancer Association Consortium (BCAC), involving 122 977 cases and 105 974 controls^18^. For Brain glioblastoma (91 cases and 218 701 controls) and Malignant neoplasm of kidney (971 cases and 217 821 controls), genetic associations were acquired from the FinnGen study^19^. Additional data for various cancers were obtained from the UK Biobank, a substantial cohort comprising 502 507 participants recruited across 22 assessment centers in the UK from 2006 to 2010^20^. Cancer cases were categorized based on ICD-9 (http://www.icd9data.com/2007/Volume1/default.htm) and ICD-10 (http://apps.who.int/classifications/icd-10/browse/2016/en), while controls were defined as individuals without any cancer code (ICD-9 or ICD-10) and no self-reported cancer diagnosis. These analyses were confined to participants of European ancestry.

It is pertinent to emphasize that all studies that contributed data to this analysis had secured the requisite institutional review board approvals within their respective countries, in strict adherence to the principles outlined in the Declaration of Helsinki.

### Genetic proxies for lipid-lowering drugs

LDL was used as a biomarker in this study because all three lipid-lowering drugs in the study were shown to reduce LDL cholesterol levels^21^. Genetic associations with LDL were sourced from the most extensive genome-wide association meta-analysis known to us, conducted by the Global Lipids Genetics Consortium^22^. This meta-analysis included ~1.3 million individuals of European ancestry. In addition, Instruments were constructed in PLINK by obtaining SNPs associated with LDL at genome-wide significance (*P*<5×10^−8^) that were in or within ±500 kb from the gene encoding each respective target (HMGCR, chromosome 5: 74632154–74657929; NPC1L1, chromosome 7: 44552134–44580914; PCSK9, chromosome 1: 55505221–55530525) using the phase 3 version 5 of the 1000 genomes project as a reference panel^23,24^. Linkage disequilibrium (LD) clumping (r2<0.001) was then applied to select nearly independent variants as genetic tools.

### MR positive control analysis

A positive control MR analysis serves to substantiate the genetic instruments of the drug by showcasing the anticipated impact on the outcome with a firmly established causal relationship to the drug under investigation^25^. Coronary atherosclerosis and coronary heart disease are thought to have strong links to LDL, where lipid-lowering drugs can have recognized benefits^26,27^. To fortify the robustness and contextualize the outcomes of our MR analysis, we conducted a supplementary examination to assess the validity of the instrument, using coronary atherosclerosis and coronary heart disease as positive control outcomes. This involved repeating the earlier MR analysis, utilizing LDL as the biomarker. Data for Coronary atherosclerosis were obtained from the FinnGen study^19^. The data on coronary heart disease were sourced from 14 genome-wide association studies on coronary artery disease conducted by Schunkert *et al*.^28^. This dataset encompasses a total of 22 233 cases and 64 762 controls, all of European descent.

### MR statistical analysis

MR analysis relies on three fundamental assumptions (Supplementary Fig 1, Supplemental Digital Content 1, http://links.lww.com/JS9/C527): (1) the genetic instrument is associated with a modifiable exposure or drug target (relevance); (2) the instrument does not share a common cause with the outcome (exchangeability); and (3) the instrument exerts no direct effect on the outcome (exclusion restriction)^29,30^.

Causal estimates were derived through inverse-variance weighted (IVW) random-effects models, with the IVW results serving as the principal reference outcomes for this study^31,32^. Additionally, to address potential limitations associated with the IVW assumption, MR Egger regression, weighted median, simple mode, and weighted mode methods were employed. The IVW method utilized multiplicative random-effects to obtain a weighted average of individual variant estimates. The resulting MR estimates were standardized to 1 SD, corresponding to an approximate 6.7 mmol/mol reduction in LDL. The mean F statistic was computed to assess the genetic instrument strength for each drug class. Consistent with convention, an F statistic exceeding 10 was indicative of minimal weak instrument bias^33^.

To address the issue of multiple testing across analyses, a Bonferroni correction was applied, setting a stringent *P*-value threshold of <0.0013 [false-positive rate=0.05/39 statistical tests (3 drug targets tested against 13 primary cancer endpoints)]. This threshold was considered indicative of ‘strong evidence’, while findings falling within the range of *P*≥0.0013 to *P*<0.05 were categorized as ‘weak evidence’. The MR analyses were conducted utilizing the R package ‘TwoSampleMR’^34,35^.

### Cell culture

Cells were cultured at 37°C and 5% CO_2_ in a humidified atmosphere. The human MCF-7 and MDAMB-231 breast cancer cell lines were procured from the Cell Resource Center of Peking Union Medical College (http://cellresource.cn), while the EMT6 murine mammary carcinoma cell line was obtained from Nanjing Cobioer Biosciences Co., Ltd. MCF-7 and MDA-MB-231 cells were cultured in Dulbecco’s Modified Eagle Medium (DMEM), while EMT6 cells were cultured in RPMI supplemented with 10% fetal bovine serum (FBS) and 1% antibiotics. Additionally, all media were supplemented with 10% FBS, 1% Glutamax, and 1% penicillin/streptomycin.

### Cell viability determination

The assessment of cell viability and cytotoxic effects induced by Lovastatin was conducted through a WST-1 assay (Abcam, ab155902). This methodology relies on the enzymatic cleavage of a stable tetrazolium salt, resulting in the formation of a soluble formazan product. Consequently, the measurement of optical density attributable to formazan serves as an indicator for cell viability, encompassing both metabolic activity and cellular respiration. Notably, each experimental iteration was replicated three times to ensure robustness and reliability of the findings.

### Apoptosis detection

Cells treated in 96-well plates were combined with Caspase-3/7 Reagent (Bio-Rad; Product Code: ICT093), following the manufacturer’s instructions, to evaluate caspase-3/7 activities. The utilization of the Caspase-3/7 assay was instrumental in assessing apoptotic activity under experimental conditions. The activation of these caspases initiates the cleavage of a substrate, generating a luminescent signal directly proportional to the extent of apoptosis.

### Detection of PD-L1 expression levels

The expression level of PD-L1 was assessed using quantitative real-time polymerase chain reaction (qRT-PCR), Western blotting (WB), and flow cytometry methods, respectively. These methods were carried out in accordance with previously described protocols and according to the manufacturer’s instructions^36–38^. The primer sequences used for PCR were as follows: GAPDH Forward: CAAGATCATCACCAATGCCT, Reverse: CCCATCACGCCACAGTTTCC; PD-L1 Forward: TGCCGACTACAAGCGAATTACTG, Reverse: CTGCTTGTCCAGATGACTTCGG. Primary antibodies targeting PD-L1 (13684, Cell Signaling Technology) and GAPDH (2118, Cell Signaling Technology) were employed. Additionally, PD-L1 Antibody (PA5-20343, Thermo Scientific) was utilized in flow cytometry analysis.

### CD8^+^ T cell-mediated cancer cell killing assay

MDA-MB-231-luciferase cells (5000 cells/well) and EMT6-luciferase cells (5000 cells/well) were seeded in 96-well plates. Human CD8^+^ T cells were isolated from human peripheral blood mononuclear cells (PBMCs), while murine CD8^+^ T cells were isolated from the spleens of healthy mice. Both human and murine CD8^+^ T cells were stimulated with T cell activators CD3/CD28 and recombinant IL-2 (10 ng/ml) for 48 h. Subsequently, the cancer cells were co-cultured with activated human CD8^+^ T cells and murine CD8^+^ T cells, followed by the addition of 100 µl of 2 mg/ml luciferin to each well.

### Animal and tumor models

All animal experiments in this study were performed in accordance with ARRIVE (Supplemental Digital Content 2, http://links.lww.com/JS9/C528) standards^39^. EMT6 cells (2×10^5 cells in 50 μl Matrigel with 50 μl PBS) were injected into the left fat pad of 5-week-old female BALB/C nude mice. Five-week-old female BALB/c nude mice aged 5 weeks were obtained from SPF Beijing Biotechnology Co. Ltd. (Beijing, China). The mice were divided into four groups: Control (Methylcellulose), Lovastatin, Paclitaxel, or a combination of Lovastatin and Paclitaxel. Treatments included vehicle, Paclitaxel (50 mg/kg, intraperitoneal), and/or Lovastatin (50 mg/kg, oral gavage) administered at 3-day intervals. Lovastatin was suspended in methylcellulose before administration. Mice were euthanized when their tumor volumes reached 1500 mm^3. Flow cytometry immune phenotyping was performed following established protocols^40,41^. On day 21, mice were euthanized, and tumor-draining lymph nodes (TDLNs) were harvested. Staining was conducted using fluorescein isothiocyanate (FITC) antimouse CD3 and efluor450 antimouse CD8 antibodies.

### Statistics

MR and bioinformatics analyses were conducted using R version 4.3.1 software. The ‘limma’ R package was utilized to identify differentially expressed genes (DEGs). Graphical data represent the mean±SD. Group comparisons were assessed using one-way ANOVA followed by Bonferroni comparisons test. Unless explicitly mentioned, significance was considered at *P*<0.05.

## Result

### MR analysis of lipid-lowering drugs and risk of 13 cancers

**Figure 2:**
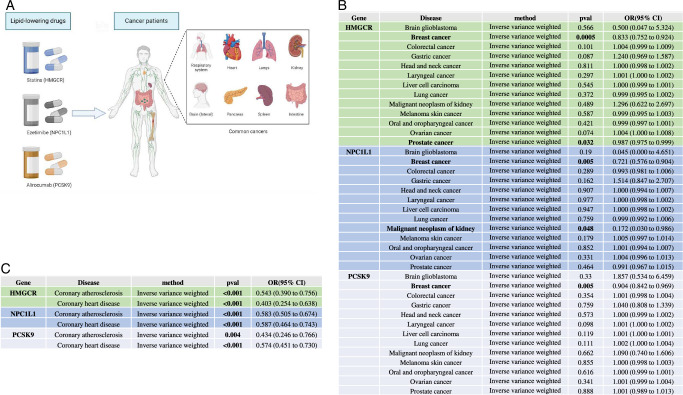
MR analysis of lipid-lowering drugs and risk of 13 cancers. (A) Schematic diagram of common cancers and genetically proxied lipid-lowering drugs. (B) Association between genetically proxied lipid-lowering drugs and risk of 13 cancer. (C) Association between genetically proxied lipid-lowering drugs as positive controls and risk of coronary artery disease. Results with *P*<0.05 were highlighted. *P*<0.0013 was considered strong evidence. A range of *P*≥0.0013 to *P*<0.05 was considered weak evidence. HMGCR indicates 3-hydroxy-3-methylglutaryl CoA reductase. LDL, low-density lipoprotein; NPC1L1, Niemann-Pick C1–like 1; OR, odds ratio; PCSK9, proprotein convertase subtilisin/Kexin type 9.

To investigate the potential therapeutic implications of lipid-lowering drugs in the context of cancer (Fig. 2A), an initial step involved the execution of MR analyses. In conducting pan-cancer MR analyses, we selected seven variants to serve as proxies for the inhibition of HMGCR, 3 for NPC1L1, and 12 for PCSK9, respectively (Supplementary Table 1, Supplemental Digital Content 3, http://links.lww.com/JS9/C529). The F statistics are all greater than 10. The complete MR analysis results of inhibition and pan-cancer of the three gene agents are presented in Supplementary Tables 2–4 (Supplemental Digital Content 4, http://links.lww.com/JS9/C530, Supplemental Digital Content 5, http://links.lww.com/JS9/C531, Supplemental Digital Content 6, http://links.lww.com/JS9/C532). The IVW analysis results are considered key results and are shown in Figure 2B.

We found that genetically proxied HMGCR inhibition was associated with breast cancer risk reduction [odds ratio (OR), 0.833 per SD reduction in LDL; 95% CI: 0.752–0.924; *P*=0.0005]. In addition, there are weak correlations between HMGCR and prostate cancer, NPC1L1 and breast cancer, malignant neoplasm of kidney, PCSK9 and breast cancer (Fig. 2B). The findings from both the *Q* test and the MR-Egger intercept test consistently indicated the absence of heterogeneity and horizontal pleiotropy in the conducted MR analysis (Supplementary Tables 5, Supplemental Digital Content 7, http://links.lww.com/JS9/C533).

To ensure the robustness of the MR analysis, supplementary positive control analyses were undertaken. Given the well-documented therapeutic efficacy of lipid-lowering drugs in mitigating coronary artery-related diseases^42^, we scrutinized the validity of the instrumental variables (IVs) by utilizing coronary artery-related disease as a positive control benchmark. Subsequent to replicating the procedural steps of previous MR analyses, our study consistently demonstrated that three genetic agents linked to lipid-lowering drugs were indeed associated with a diminished risk of coronary artery-related disease (Fig. 2C). This result validated the accuracy of our MR analysis.

In conclusion, the results of MR suggest that statin use is associated with better survival in breast cancer patients.

### Lovastatin significantly inhibits paclitaxel-induced PD-L1 expression and assists in its inhibition of tumor cell growth

The MR findings indicate a potential association between statins and improved prognosis in breast cancer patients. To delve deeper into the potential role of statins in breast cancer, lovastatin, a widely used fat-soluble statin, was chosen as the representative statin for this investigation. Previous studies within the context of breast cancer have consistently shown that fat-soluble statins, including lovastatin, tend to display more pronounced effects compared to their water-soluble counterparts^43^. We first studied the effects of lovastatin on breast cancer cells in *vitro*. MCF-7 and MDA-MB-231 served as in *vitro* cell models for breast cancer. Treatment with 5, 10, and 20 μM concentrations of lovastatin resulted in a significant decrease in cell viability of MDA-MB-231 and MCF-7 cells after 24 and 48 h (Supplementary Figs 2A, B, Supplemental Digital Content 8, http://links.lww.com/JS9/C534). Furthermore, lovastatin increases caspase-3/7 activity in a dose-dependent manner. Caspase-3/7 was increased approximately fourfold after induction with 20 μM lovastatin for 48 h in MDA-MB-231 and threefold in MCF-7 (Supplementary Figs 3A, 3B, Supplemental Digital Content 9, http://links.lww.com/JS9/C535).

**Figure 3:**
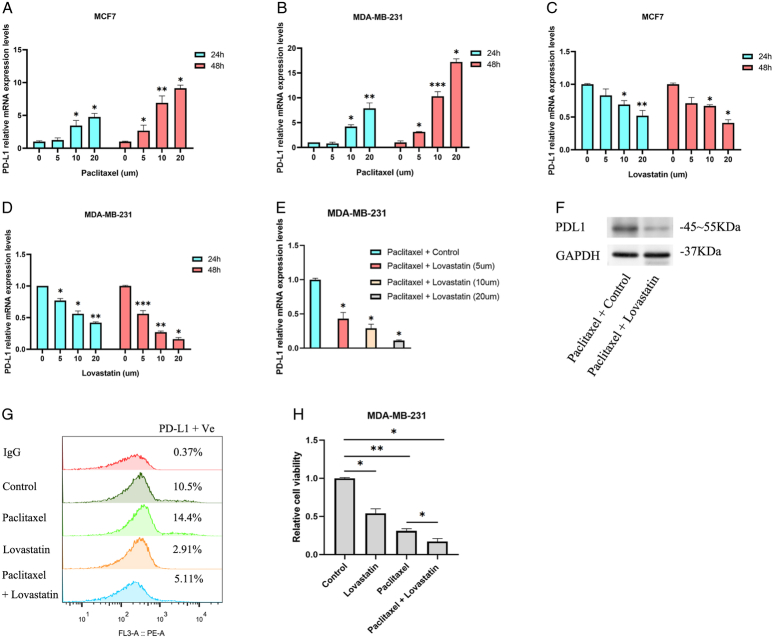
Lovastatin significantly inhibits paclitaxel-induced PD-L1 expression and assists in its inhibition of tumor cell growth. (A) The relative changes in mRNA expression levels of PD-L1 after treatment of MCF-7 cells with different concentrations of paclitaxel. (B) The relative changes in mRNA expression levels of PD-L1 after treatment of MDA-MB-231 cells with different concentrations of paclitaxel. (C) The relative changes in mRNA expression levels of PD-L1 after treatment of MCF-7 cells with different concentrations of lovastatin. (D) The relative changes in mRNA expression levels of PD-L1 after treatment of MDA-MB-231 cells with different concentrations of lovastatin. (E) MDA-MB-231 cells were first treated with paclitaxel (20 μm), and then treated with different concentrations of lovastatin after 24 h, and then their PD-L1 expression levels were detected. (F) MDA-MB-231 cells were first treated with paclitaxel (20 μM) and then treated with lovastatin (20 μM) 24 h later. The protein expression level of PD-L1 in MDA-MB-231 cells decreased significantly. (G) The effects of different treatments [paclitaxel (20 μM) or lovastatin (20 μM)] on the expression level of PD-L1 on the surface of MDA-MB-231 cells were detected by flow cytometry. (H) Effects of different treatments [paclitaxel (20 μM) or lovastatin (20 μM)] on relative cell viability of MDA-MB-231 cells. *, *P*<0.05; **, *P*<0.01; and ***, *P*<0.001.

Paclitaxel stands as a frequently employed chemotherapeutic agent in the management of breast cancer patients. Nevertheless, a notable drawback of paclitaxel treatment is its propensity to induce overexpression of programmed death-ligand 1 (PD-L1) in breast cancer cases, thereby presenting a challenge to effective patient care. Building upon the reported potential of statins to inhibit PD-L1 expression^38,44^, our objective was to explore the feasibility of utilizing statins as viable adjuvant agents in paclitaxel treatment strategies for breast cancer. Following treatment with varying concentrations of paclitaxel, the mRNA expression levels of PD-L1 were assessed 24 and 48 h post-treatment. The outcomes revealed a significant upregulation of PD-L1 expression in breast cancer cells in response to paclitaxel treatment (Figs 3A, B). Consistent with previous research results^38,44^, lovastatin can significantly inhibit PD-L1 expression in breast cancer cells (Figs 3C, D).

We conducted further investigations to ascertain whether lovastatin could inhibit paclitaxel-induced PD-L1 expression. Based on previous findings, MDA-MB-231 is more sensitive to lovastatin. MDA-MB-231 was used as a cell model for further studies. PCR results revealed that lovastatin exhibited a dose-dependent inhibitory effect on the expression of PD-L1 mRNA induced by paclitaxel (Fig. 3E). PD-L1 protein expression level was also significantly inhibited by lovastatin (Fig. 3F). In addition, we explored the effects of lovastatin and paclitaxel on PD-L1 expression in breast cancer cells by flow cytometry. Consistent with our previous results that paclitaxel induces PD-L1 expression in cells and lovastatin inhibits PD-L1 expression in cells, the combination of lovastatin and paclitaxel significantly inhibited inhibition of paclitaxel-induced PD-L1 expression (Fig. 3G). An additional noteworthy discovery is that the inhibitory effects of both paclitaxel and lovastatin on the viability of breast cancer cells exhibit an additive nature (Fig. 3H). The concurrent use of paclitaxel and lovastatin results in a synergistic enhancement of inhibitory effects on breast cancer cells, thereby achieving a more pronounced impact.

### Lovastatin and paclitaxel combination therapy enhances tumor killing capacity of CD8^+^ T cells

Expression profiling data of breast tumors in lovastatin-treated mice were obtained from the GEO database (GSE42787)^45^. Lovastatin treatment upregulated 39 genes and downregulated 49 genes compared to controls (Supplementary Table 6, Supplemental Digital Content 10, http://links.lww.com/JS9/C536). The functional enrichment results surface that lovastatin in breast cancer may be associated with pathways such as Interferon alpha/beta signaling, response to hypoxia, angiogenesis, and immune response (Supplementary Fig 4, Supplemental Digital Content 11, http://links.lww.com/JS9/C537).

**Figure 4:**
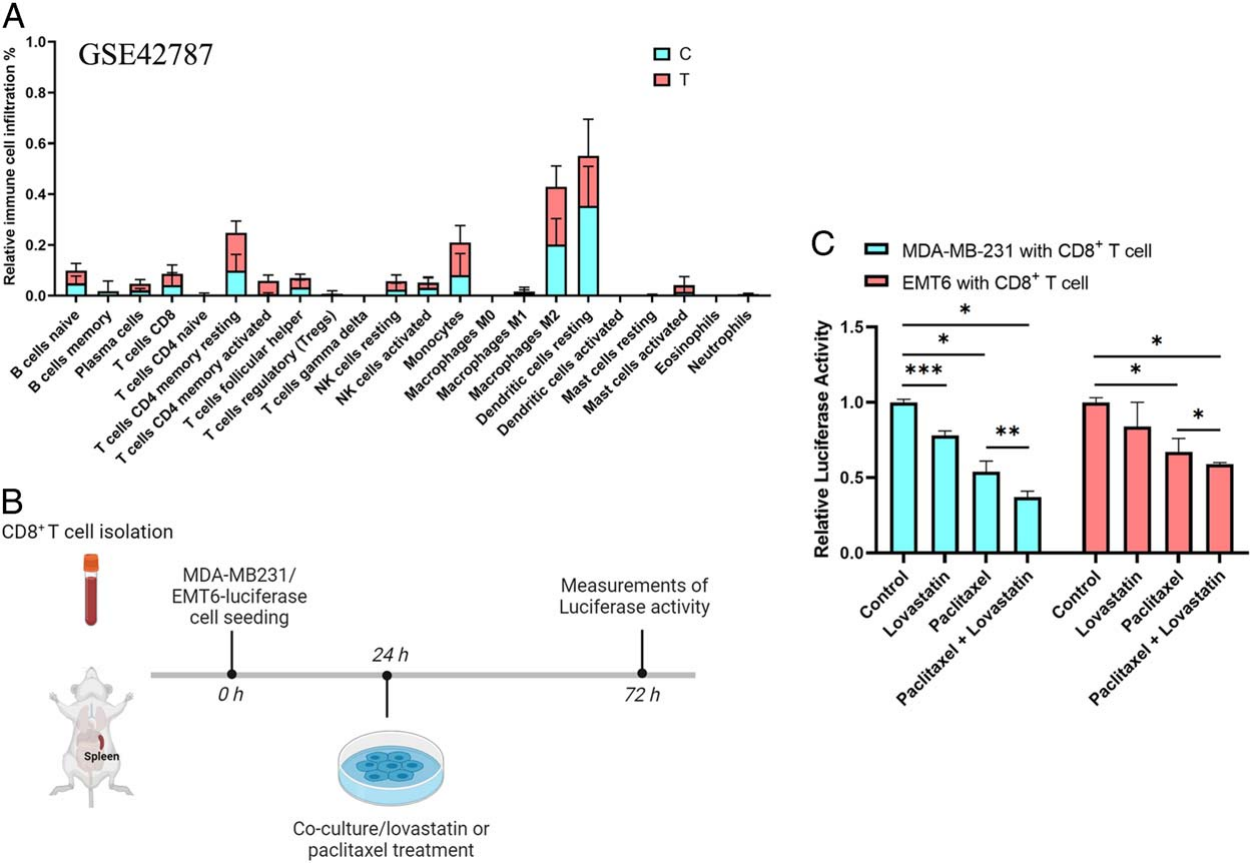
Lovastatin and paclitaxel combination therapy enhances tumor killing capacity of CD8+ T cells. (A) Effect of lovastatin treatment on immune cell infiltration, data from GSE42787, prediction method based on CIBERSORT. (B) Experimental design of a co-culture system for the detection of CD8^+^ T cell-mediated cytotoxicity. (C) PBMC and mice CD8^+^ T cell-mediated cytotoxicity in MDA-MB-231- and EMT6-luciferase cells following the indicated treatments (*n*=3). *, *P*<0.05; **, *P*<0.01; and ***, *P*<0.001.

In this study, we focused on tumor immunity, therefore, we further analyzed the effect of lovastatin treatment on immune cell infiltration, and the results showed that lovastatin significantly enhanced immune cell infiltration in tumor cells, especially in T cells (Fig. 4A).

The ability of PD-L1 to inhibit cytotoxic CD8^+^ T cells is a key adaptive mechanism employed by PD-L1-expressing cells to suppress the immune system^46,47^. In the study in Figure 3, our results showed that lovastatin inhibited PD-L1 expression in breast cancer cells. Thus, lovastatin may have the potential to promote tumor killing by CD8^+^ T cells. MDA-MB-231- and EMT6-luciferase cells were co-cultured with CD8^+^ T cells isolated from human PBMC and mice spleen, respectively (Fig. 4B). After activation of T cells with anti-CD3 and CD28, they were treated with lovastatin and paclitaxel, and then their luciferase activities were measured after 48 h. The results showed that the cytotoxic effect of CD8^+^ T cells was enhanced by the combination of lovastatin and paclitaxel compared to paclitaxel alone (Fig. 4C).

### Lovastatin and paclitaxel combination therapy enhances CD8^+^ T cell infiltration and improves therapeutic efficacy in *vivo*

**Figure 5:**
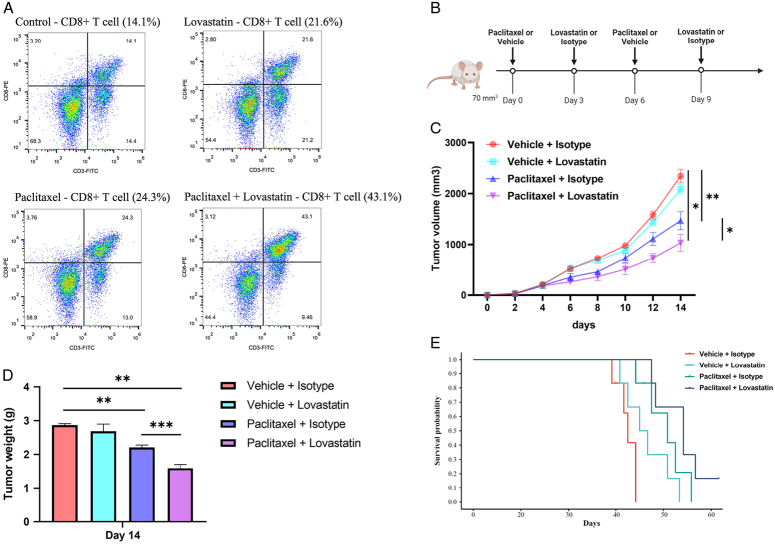
Lovastatin and paclitaxel combination therapy enhances CD8^+^ T cell infiltration and improves therapeutic efficacy in *vivo*. (A) Flow cytometry analysis of T lymphocytes from draining lymph nodes (DLNs) isolated from mice carrying EMT6 was performed to detect the proportion of CD8^+^ T cells. (B) Schematic representation of mice treated with paclitaxel and lovastatin. Control group received vehicle and isotype. (C) Volume measurement of mammary tumors over time in mice transplanted with EMT6 cells and treated according to the regimen described in B. (D) Weight of tumors isolated at humane endpoint post-treatment (day 14). (E) Survival analysis of mice with different treatments (*n*=6). *, *P*<0.05; **, *P*<0.01; and ***, *P*<0.001.

An analysis of T lymphocytes in draining lymph nodes (DLN) isolated from mice carrying EMT6 was performed by flow cytometry. The proportion of CD8^+^ T cell populations was significantly increased in mice treated with a combination of lovastatin and paclitaxel (Fig. 5A).

These in *vitro* data prompted us to further investigate the antitumor effects of lovastatin in combination with paclitaxel in mice models. At first, mice were treated with paclitaxel (to induce PD-L1) and then with lovastatin, as shown in Figure 5B. Both compounds were administered when mammary tumors reached a palpable and measurable volume (70 mm^3^). Hormonal mice were monitored until they reached a humane endpoint (tumor volume ≤2 cm^3^). Treatment of mice with lovastatin in combination with paclitaxel significantly reduced tumor volume (Fig. 5C) and weight (Fig. 5D) compared with control mice and was more effective than paclitaxel or lovastatin alone. Notably, survival was higher with combination therapy compared with treatment with lovastatin or paclitaxel alone (Fig. 5E).

## Discussion

In recent years, immunotherapy has become increasingly prominent in the clinical treatment of various cancers, including breast cancer^48^. However, a subset of patients demonstrates limited responsiveness or acquires resistance to immunotherapeutic interventions. Therefore, there is a pressing need for novel therapeutic approaches to augment the efficacy of immunotherapy. PD-L1 emerges as a crucial therapeutic target and prognostic indicator in cancer treatment, highlighting its significant clinical importance^49^. Nonetheless, it is important to acknowledge that certain breast cancer patients may harbor ‘cold’ tumors that exhibit reduced responsiveness to immunotherapy.

Cholesterol serves as a crucial constituent of mammalian cell membranes, particularly essential for highly proliferative cells such as cancer cells, which necessitate heightened cholesterol levels to support rapid membrane synthesis^50–52^. Statins may exert potential anticancer effects by modulating tumor proliferation through the inhibition of intracellular cholesterol biosynthesis^53^. Despite ambiguity in epidemiological evidence concerning the impact of statin use on cancer patients, several observational studies imply a potential inhibitory effect of statins on tumor progression. However, these studies are susceptible to confounding variables, selection bias, and other factors that may compromise the validity of their findings. MR analysis leverages genetic variations as IVs to enhance causal inference in the exposure-outcome relationship, thereby bolstering the reliability of study results. Therefore, in this study, we first analyzed the potential risk relationship between lipid-lowering drugs and cancer risk by MR, and the results showed that statin use was associated with a better prognosis in breast cancer patients.

Statins have been shown to modulate the mevalonate pathway, exerting inhibitory effects on breast cancer cell proliferation by targeting HMGCR, a protein markedly upregulated in breast cancer patients^54^. Additionally, there is evidence suggesting that statins may possess the capability to suppress PD-L1 expression^55^. Presently, paclitaxel stands as one of the primary treatments for breast cancer, yet its efficacy varies, with recurrent cases being common^56,57^. The presence of PD-L1 in both tumors and tumor-infiltrating cells among breast cancer patients indicates that blocking PD-L1 could augment the anticancer properties of paclitaxel analogs. Consequently, we sought to investigate whether statins could serve as adjunctive therapies to paclitaxel. In our in *vitro* experiments, we observed that lovastatin significantly attenuated paclitaxel-induced PD-L1 expression and facilitated the inhibition of tumor cell growth. Further studies suggest that lovastatin may have the potential to promote tumor killing by CD8^+^ T cells, and that the combination of lovastatin and paclitaxel enhanced the cytotoxicity of CD8^+^ T cells compared to paclitaxel alone. Yulian *et al*.^58^ reported the results of a clinical trial suggesting that the combination of statins with fluorouracil, adriamycin, and cyclophosphamide may better improve the prognosis of patients with locally advanced breast cancer. However, studies combining statins with paclitaxel in breast cancer remain scarce. Our in *vivo* study shows for the first time that the combination of statins and paclitaxel can increase CD8^+^ T cell infiltration resulting in better prognostic characteristics in mice.

It is noteworthy that over a quarter of all 40-year-old Americans have used statins, yet their use may pose potential risks (such as affecting glucose homeostasis, cognitive function, renal and hepatic function, increasing the risk of hemorrhagic stroke, and cataracts) to certain high-risk patients^59,60^. Therefore, caution should be exercised in prescribing statins to high-risk patients. Additionally, statin use has been suggested to potentially play a role in cancer prevention (including breast cancer, prostate cancer, pancreatic cancer, and colon cancer), possibly linked to cholesterol-mediated tumorigenesis^61–64^. However, this remains controversial and requires further evidence. Although most epidemiological and preclinical studies have observed statin use to be associated with improved prognosis and significant risk reduction in cancer patients, these factors alone are unlikely to fully explain the observed beneficial effects. This study specifically focuses on breast cancer and elucidates the potential role of statins in this context. In summary, the findings of this study suggest that statin use is linked to a decreased risk of breast cancer and may have potential as an adjuvant therapy for breast cancer treatment.

### Limitations of the study

This study has certain limitations that require further investigation to enhance its clinical significance. Specifically, the effective dosage of statins utilized in our in vitro experiments exceeded concentrations typically observed in human plasma, while for our in vivo experiments in mice, a dosage of 50 mg/kg was selected based on available literature^65,66^. However, early studies suggest that mice metabolize statins at a considerably faster rate than humans due to the rapid upregulation of their HMG-CoA reductase post-treatment, emphasizing the need for dose adjustments in human experiments^67,68^. The potential relationship between statins and cancer prevention, particularly their role in tumorigenesis, was not investigated in this study and will be the focus of our future research. Additionally, this study concentrates on the role of statins in the adjuvant treatment of breast cancer. Further investigation into the potential role of statins in other types of cancer is warranted.

## Ethical approval

All animal experiments were conducted in accordance with protocols approved by the Animal Care and Use Committee of the Second Affiliated Hospital of Guangxi Medical University [Approval number: 2023-KY (0906)].

## Patient consent for publication

Not applicable.

Consent for publication: All the authors have approved the manuscript.

## Sources of funding

This work was supported by grants from the Guangxi Natural Science Foundation (No. 2023GXNSFBA026137).

## Author contribution

K.Q. and S.N.: designed the overall study and revised the manuscript; K.Q., S.Z., Y.P., W.T., and R.H.: completed most of the experiments for this study; W.T. and L.L.: collected and processed raw data; L.L., X.L., Y.P., and S.G.: analyzed data and drafted the manuscript.

## Conflicts of interest disclosure

The authors declare that there are no competing interests associated with the manuscript.

## Research registration unique identifying number (UIN)

1. Name of the registry: not applicable.
2. Unique identifying number or registration ID: not applicable.
3. Hyperlink to your specific registration (must be publicly accessible and will be checked): not applicable.

## Guarantor

Kun Qiao and Shipeng Ning.

## Data availability statement

Data are available on reasonable request. The datasets used and/or analyzed during the current study are available from the corresponding author on reasonable request.

## Provenance and peer review

“Not commissioned, externally peer-reviewed”.

## Supplementary Material

## References

[1] Mattiuzzi C Lippi G . Current cancer epidemiology. J Epidemiol Glob Health 2019;9:217–222. 31854162 10.2991/jegh.k.191008.001 PMC7310786

[2] Siegel RL Miller KD Jemal A . Cancer statistics, 2018. CA Cancer J Clin 2018;68:7–30. 29313949 10.3322/caac.21442

[3] Nagaraju GP Malla RR Basha R . Contemporary clinical trials in pancreatic cancer immunotherapy targeting PD-1 and PD-L1. Semin Cancer Biol 2022;86(Pt 3):616–621. 34774995 10.1016/j.semcancer.2021.11.003

[4] Wang Y Zhou S Yang F . Treatment-related adverse events of PD-1 and PD-L1 inhibitors in clinical trials: a systematic review and meta-analysis. JAMA Oncol 2019;5:1008–1019. 31021376 10.1001/jamaoncol.2019.0393 PMC6487913

[5] Jhunjhunwala S Hammer C Delamarre L . Antigen presentation in cancer: insights into tumour immunogenicity and immune evasion. Nat Rev Cancer 2021;21:298–312. 33750922 10.1038/s41568-021-00339-z

[6] Schairer C Freedman DM Gadalla SM . Lipid-lowering drugs, dyslipidemia, and breast cancer risk in a Medicare population. Breast Cancer Res Treat 2018;169:607–614. 29450675 10.1007/s10549-018-4680-7 PMC6705395

[7] Vallianou NG Kostantinou A Kougias M . Statins and cancer. Anticancer Agents Med Chem 2014;14:706–712. 24295174 10.2174/1871520613666131129105035

[8] Jiang W Hu JW He XR . Statins: a repurposed drug to fight cancer. J Exp Clin Cancer Res 2021;40:241. 34303383 10.1186/s13046-021-02041-2 PMC8306262

[9] Duarte JA de Barros ALB Leite EA . The potential use of simvastatin for cancer treatment: A review. Biomed Pharmacother 2021;141:111858. 34323700 10.1016/j.biopha.2021.111858

[10] Nielsen SF Nordestgaard BG Bojesen SE . Statin use and reduced cancer-related mortality. N Engl J Med 2012;367:1792–1802. 23134381 10.1056/NEJMoa1201735

[11] Lim WJ Lee M Oh Y . Statins decrease programmed death-ligand 1 (PD-L1) by inhibiting AKT and β-catenin signaling. Cells 2021;10:2488. 34572136 10.3390/cells10092488 PMC8472538

[12] Mao W Cai Y Chen D . Statin shapes inflamed tumor microenvironment and enhances immune checkpoint blockade in non-small cell lung cancer. JCI Insight 2022;7:e161940. 35943796 10.1172/jci.insight.161940 PMC9675559

[13] Uemura N Hayashi H Liu Z . Statins exert anti-growth effects by suppressing YAP/TAZ expressions via JNK signal activation and eliminate the immune suppression by downregulating PD-L1 expression in pancreatic cancer. Am J Cancer Res 2023;13:2041–2054. 37293171 PMC10244110

[14] Zheng X Cui XX Gao Z . Atorvastatin and celecoxib in combination inhibits the progression of androgen-dependent LNCaP xenograft prostate tumors to androgen independence. Cancer Prev Res (Phila) 2010;3:114–124. 20051379 10.1158/1940-6207.CAPR-09-0059 PMC2803700

[15] Ghosh-Choudhury N Mandal CC Ghosh-Choudhury N . Simvastatin induces derepression of PTEN expression via NFkappaB to inhibit breast cancer cell growth. Cell Signal 2010;22:749–758. 20060890 10.1016/j.cellsig.2009.12.010 PMC2826504

[16] Davey Smith G Hemani G . Mendelian randomization: genetic anchors for causal inference in epidemiological studies. Hum Mol Genet 2014;23(R1):R89–R98. 25064373 10.1093/hmg/ddu328 PMC4170722

[17] Zhang Z Li L Wu J . A Mendelian randomization-based approach to explore the relationship between leukocyte counts and breast cancer risk in European ethnic groups. Sci Rep 2023;13:16979. 37813992 10.1038/s41598-023-44397-9 PMC10562486

[18] Michailidou K Lindström S Dennis J . Association analysis identifies 65 new breast cancer risk loci. Nature 2017;551:92–94. 29059683 10.1038/nature24284 PMC5798588

[19] FINNGEN. FinnGen: FinnGen documentation of R6 release. 2022 https://finngen.gitbook.io/documentation/

[20] Sudlow C Gallacher J Allen N . UK biobank: an open access resource for identifying the causes of a wide range of complex diseases of middle and old age. PLoS Med 2015;12:e1001779. 25826379 10.1371/journal.pmed.1001779 PMC4380465

[21] Zhao SS Yiu ZZN Barton A . Association of lipid-lowering drugs with risk of psoriasis: a Mendelian randomization study. JAMA Dermatol 2023;159:275–280. 36696131 10.1001/jamadermatol.2022.6051 PMC9878432

[22] Chang CC Chow CC Tellier LC . Second-generation PLINK: rising to the challenge of larger and richer datasets. Gigascience 2015;4:7. 25722852 10.1186/s13742-015-0047-8 PMC4342193

[23] 1000 Genomes Project Consortium Auton A Brooks LD Durbin RM . A global reference for human genetic variation. Nature 2015;526:68–74. 26432245 10.1038/nature15393 PMC4750478

[24] Li Z Zhang B Liu Q . Genetic association of lipids and lipid-lowering drug target genes with non-alcoholic fatty liver disease. EBioMedicine 2023;90:104543. 37002989 10.1016/j.ebiom.2023.104543 PMC10070091

[25] Burgess S Davey Smith G Davies NM . Guidelines for performing Mendelian randomization investigations: update for summer 2023. Wellcome Open Res 2023;4:186. 32760811 10.12688/wellcomeopenres.15555.1 PMC7384151

[26] Ference BA Ginsberg HN Graham I . Low-density lipoproteins cause atherosclerotic cardiovascular disease. 1. Evidence from genetic, epidemiologic, and clinical studies. A consensus statement from the European Atherosclerosis Society Consensus Panel. Eur Heart J 2017;38:2459–2472. 28444290 10.1093/eurheartj/ehx144 PMC5837225

[27] Wang HH Garruti G Liu M . Cholesterol and lipoprotein metabolism and atherosclerosis: recent advances in reverse cholesterol transport. Ann Hepatol 2017;16(suppl. 1: s3-105):s27–s42. 29080338 10.5604/01.3001.0010.5495

[28] Schunkert H König IR Kathiresan S . Large-scale association analysis identifies 13 new susceptibility loci for coronary artery disease. Nat Genet 2011;43:333–338. 21378990 10.1038/ng.784 PMC3119261

[29] van Kippersluis H Rietveld CA . Pleiotropy-robust Mendelian randomization. Int J Epidemiol 2018;47:1279–1288. 28338774 10.1093/ije/dyx002 PMC6124631

[30] Schmidt AF Finan C Gordillo-Marañón M . Genetic drug target validation using Mendelian randomisation. Nat Commun 2020;11:3255. 32591531 10.1038/s41467-020-16969-0 PMC7320010

[31] Yu H Wan X Yang M . A large-scale causal analysis of gut microbiota and delirium: a Mendelian randomization study. J Affect Disord 2023;329:64–71. 36842654 10.1016/j.jad.2023.02.078

[32] Cho Y Haycock PC Sanderson E . Exploiting horizontal pleiotropy to search for causal pathways within a Mendelian randomization framework. Nat Commun 2020;11:1010. 32081875 10.1038/s41467-020-14452-4 PMC7035387

[33] Yarmolinsky J Bouras E Constantinescu A . PRACTICAL consortium; VA Million Veteran Program; Gill D, Tsilidis KK. Genetically proxied glucose-lowering drug target perturbation and risk of cancer: a Mendelian randomisation analysis. Diabetologia 2023;66:1481–1500. 37171501 10.1007/s00125-023-05925-4 PMC10317892

[34] Hemani G Zheng J Elsworth B . The MR-Base platform supports systematic causal inference across the human phenome. Elife 2018;7:e34408. 29846171 10.7554/eLife.34408 PMC5976434

[35] Xue H Shen X Pan W . Constrained maximum likelihood-based Mendelian randomization robust to both correlated and uncorrelated pleiotropic effects. Am J Hum Genet 2021;108:1251–1269. 34214446 10.1016/j.ajhg.2021.05.014 PMC8322939

[36] Saito A Tojo M Kumagai Y . Flow cytometry detection of cell type-specific expression of programmed death receptor ligand-1 (PD-L1) in colorectal cancer specimens. Heliyon 2020;7:e05880. 33458446 10.1016/j.heliyon.2020.e05880 PMC7797507

[37] Li M Shang H Wang T . Huanglian decoction suppresses the growth of hepatocellular carcinoma cells by reducing CCNB1 expression. World J Gastroenterol 2021;27:939–958. 33776365 10.3748/wjg.v27.i10.939 PMC7968131

[38] Majidi M Safaee S Amini M . The effects of chemotherapeutic drugs on PD-L1 gene expression in breast cancer cell lines. Med Oncol 2021;38:147. 34687372 10.1007/s12032-021-01556-0

[39] Kilkenny C Browne WJ Cuthill IC . Improving bioscience research reporting: the ARRIVE guidelines for reporting animal research. PLoS Biol 2010;8:e1000412. 20613859 10.1371/journal.pbio.1000412 PMC2893951

[40] Im EJ Lee CH Moon PG . Sulfisoxazole inhibits the secretion of small extracellular vesicles by targeting the endothelin receptor A. Nat Commun 2019;10:1387. 30918259 10.1038/s41467-019-09387-4 PMC6437193

[41] Pockley AG Foulds GA Oughton JA . Immune Cell Phenotyping Using Flow Cytometry. Curr Protoc Toxicol 2015;66:18.8.1–18.8.34. 10.1002/0471140856.tx1808s66 26523471

[42] Almeida SO Budoff M . Effect of statins on atherosclerotic plaque. Trends Cardiovasc Med 2019;29:451–455. 30642643 10.1016/j.tcm.2019.01.001

[43] Beckwitt CH Shiraha K Wells A . Lipophilic statins limit cancer cell growth and survival, via involvement of Akt signaling. PLoS One 2018;13:e0197422. 29763460 10.1371/journal.pone.0197422 PMC5953490

[44] Yang Q Shi G Chen X . Nanomicelle protects the immune activation effects of Paclitaxel and sensitizes tumors to anti-PD-1 Immunotherapy. Theranostics 2020;10:8382–8399. 32724476 10.7150/thno.45391 PMC7381738

[45] Mira E Carmona-Rodríguez L Tardáguila M . A lovastatin-elicited genetic program inhibits M2 macrophage polarization and enhances T cell infiltration into spontaneous mouse mammary tumors. Oncotarget 2013;4:2288–2301. 24317954 10.18632/oncotarget.1376 PMC3926827

[46] Raskov H Orhan A Christensen JP . Cytotoxic CD8+ T cells in cancer and cancer immunotherapy. Br J Cancer 2021;124:359–367. 32929195 10.1038/s41416-020-01048-4 PMC7853123

[47] Sun Y Tan J Miao Y . The role of PD-L1 in the immune dysfunction that mediates hypoxia-induced multiple organ injury. Cell Commun Signal 2021;19:76. 34256773 10.1186/s12964-021-00742-x PMC8276205

[48] Liu Y Hu Y Xue J . Advances in immunotherapy for triple-negative breast cancer. Mol Cancer 2023;22:145. 37660039 10.1186/s12943-023-01850-7 PMC10474743

[49] Wan W Ao X Chen Q . METTL3/IGF2BP3 axis inhibits tumor immune surveillance by upregulating N6-methyladenosine modification of PD-L1 mRNA in breast cancer. Mol Cancer 2022;21:60. 35197058 10.1186/s12943-021-01447-y PMC8864846

[50] Munir MT Ponce C Powell CA . The contribution of cholesterol and epigenetic changes to the pathophysiology of breast cancer. J Steroid Biochem Mol Biol 2018;183:1–9. 29733910 10.1016/j.jsbmb.2018.05.001

[51] Centonze G Natalini D Piccolantonio A . Cholesterol and its derivatives: multifaceted players in breast cancer progression. Front Oncol 2022;12:906670. 35719918 10.3389/fonc.2022.906670 PMC9204587

[52] Wang Y Zhou X Lei Y . NNMT contributes to high metastasis of triple negative breast cancer by enhancing PP2A/MEK/ERK/c-Jun/ABCA1 pathway mediated membrane fluidity. Cancer Lett 2022;547:215884. 35988817 10.1016/j.canlet.2022.215884

[53] Wang A Wakelee HA Aragaki AK . Protective effects of statins in cancer: should they be prescribed for high-risk patients? Curr Atheroscler Rep 2016;18:72. 27796821 10.1007/s11883-016-0625-y

[54] Bjarnadottir O Romero Q Bendahl PO . Targeting HMG-CoA reductase with statins in a window-of-opportunity breast cancer trial. Breast Cancer Res Treat 2013;138:499–508. 23471651 10.1007/s10549-013-2473-6

[55] Choe EJ Lee CH Bae JH . Atorvastatin enhances the efficacy of immune checkpoint therapy and suppresses the cellular and extracellular vesicle PD-L1. Pharmaceutics 2022;14:1660. 36015287 10.3390/pharmaceutics14081660 PMC9414447

[56] Schmid P Adams S Rugo HS . IMpassion130 trial investigators. atezolizumab and nab-paclitaxel in advanced triple-negative breast cancer. N Engl J Med 2018;379:2108–2121. 30345906 10.1056/NEJMoa1809615

[57] Abu Samaan TM Samec M Liskova A . Paclitaxel’s mechanistic and clinical effects on breast cancer. Biomolecules 2019;9:789. 31783552 10.3390/biom9120789 PMC6995578

[58] Yulian ED Siregar NC Bajuadji . Combination of simvastatin and FAC improves response to neoadjuvant chemotherapy in locally advanced breast cancer. Cancer Res Treat 2021;53:1072–1083. 33705623 10.4143/crt.2020.1024 PMC8524017

[59] Mach F Ray KK Wiklund O . European Atherosclerosis Society Consensus Panel. Adverse effects of statin therapy: perception vs. the evidence - focus on glucose homeostasis, cognitive, renal and hepatic function, haemorrhagic stroke and cataract. Eur Heart J 2018;39:2526–2539. 29718253 10.1093/eurheartj/ehy182 PMC6047411

[60] Kostapanos MS Milionis HJ Elisaf MS . Rosuvastatin-associated adverse effects and drug-drug interactions in the clinical setting of dyslipidemia. Am J Cardiovasc Drugs 2010;10:11–28. 20104931 10.2165/13168600-000000000-00000

[61] Alfaqih MA Allott EH Hamilton RJ . The current evidence on statin use and prostate cancer prevention: are we there yet? Nat Rev Urol 2017;14:107–119. 27779230 10.1038/nrurol.2016.199 PMC5830185

[62] Min Y Wei X Liu Z . Assessing the role of lipid-lowering therapy on multi-cancer prevention: a mendelian randomization study. Front Pharmacol 2023;14:1109580. 37153802 10.3389/fphar.2023.1109580 PMC10154601

[63] Saito K Sato Y Nakatani E . Statin exposure and pancreatic cancer incidence: a Japanese regional population-based cohort study, the Shizuoka Study. Cancer Prev Res (Phila) 2021;14:863–872. 34244151 10.1158/1940-6207.CAPR-21-0123

[64] Han JX Tao ZH Wang JL . Microbiota-derived tryptophan catabolites mediate the chemopreventive effects of statins on colorectal cancer. Nat Microbiol 2023;8:919–933. 37069401 10.1038/s41564-023-01363-5

[65] Minz AP Mohapatra D Dutta M . Statins abrogate gemcitabine-induced PD-L1 expression in pancreatic cancer-associated fibroblasts and cancer cells with improved therapeutic outcome. Cancer Immunol Immunother 2023;72:4261–4278. 37926727 10.1007/s00262-023-03562-9 PMC10992415

[66] Jamil A Aamir Mirza M Anwer MK . Co-delivery of gemcitabine and simvastatin through PLGA polymeric nanoparticles for the treatment of pancreatic cancer: in-vitro characterization, cellular uptake, and pharmacokinetic studies. Drug Dev Ind Pharm 2019;45:745–753. 30632800 10.1080/03639045.2019.1569040

[67] Agarwal P Rashighi M Essien KI . Simvastatin prevents and reverses depigmentation in a mouse model of vitiligo. J Invest Dermatol 2015;135:1080–1088. 25521459 10.1038/jid.2014.529 PMC4366328

[68] Kita T Brown MS Goldstein JL . Feedback regulation of 3-hydroxy-3-methylglutaryl coenzyme A reductase in livers of mice treated with mevinolin, a competitive inhibitor of the reductase. J Clin Invest 1980;66:1094–1100. 6903572 10.1172/JCI109938 PMC371547

