# Supplementary material for: Statins inhibit paclitaxel-induced PD-L1 expression and increase CD8+ T cytotoxicity for better prognosis in breast cancer
Source: Int J Surg. 2024 May 13;110(8):4716–26. doi: 10.1097/JS9.0000000000001582 (PMC11325938; doi:10.1097/JS9.0000000000001582)
Supplement: Supplementary file 1 [file js9-110-4716-s001.pdf]

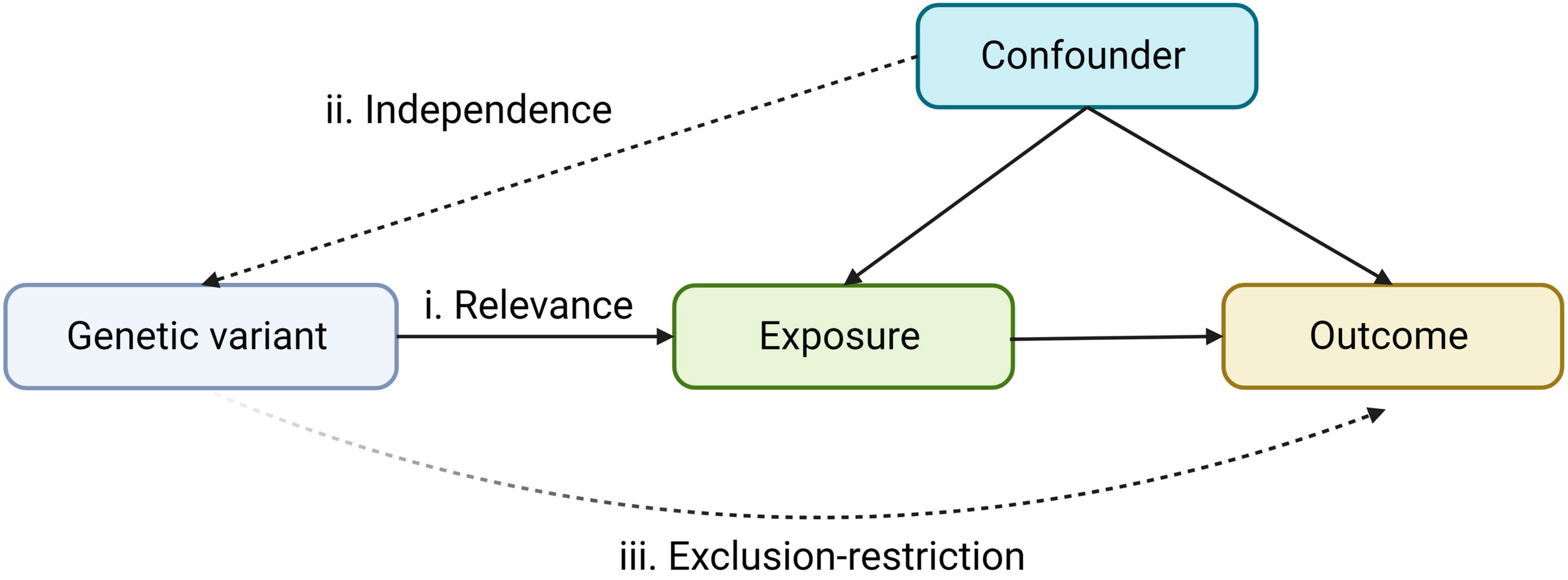

Supplementary Figure 1. Illustration of instrumental variable assumptions. Mendelian randomization (MR) provides evidence about putative causal relationships between modifiable exposures and disease outcomes, using genetic variants that are associated with exposure variation at a population level. A genetic variant can be considered as a valid instrumental variable for an exposure if it satisfies the instrumental variable assumptions: it is associated with the exposure in a specific way (assumption 1) that does not affect the outcome except via the exposure (assumption 3), and it is not associated with the outcome due to confounding (assumption 2).
