# Supplementary material for: Statins inhibit paclitaxel-induced PD-L1 expression and increase CD8+ T cytotoxicity for better prognosis in breast cancer
Source: Int J Surg. 2024 May 13;110(8):4716–26. doi: 10.1097/JS9.0000000000001582 (PMC11325938; doi:10.1097/JS9.0000000000001582)
Supplement: Supplementary file 8 [file js9-110-4716-s008.pdf]

A

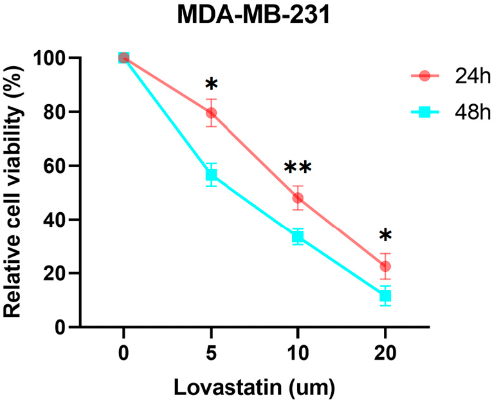

B

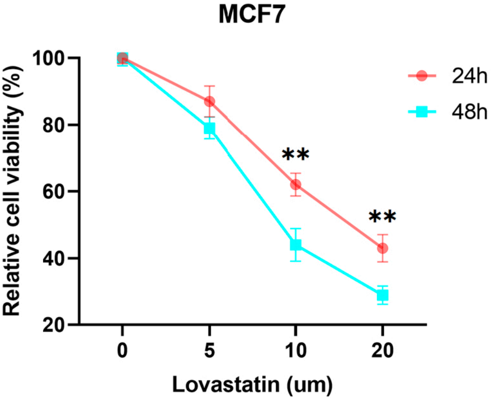

Supplementary Figure 2. Effects of lovastatin treatment with different concentration gradients on cell viability of MDA-MB-231 (A) and MCF-7 (B) cells. *, $p < 0.05$; **, $p < 0.01$; and ***, $p < 0.001$.
