# Supplementary material for: Statins inhibit paclitaxel-induced PD-L1 expression and increase CD8+ T cytotoxicity for better prognosis in breast cancer
Source: Int J Surg. 2024 May 13;110(8):4716–26. doi: 10.1097/JS9.0000000000001582 (PMC11325938; doi:10.1097/JS9.0000000000001582)
Supplement: Supplementary file 9 [file js9-110-4716-s009.pdf]

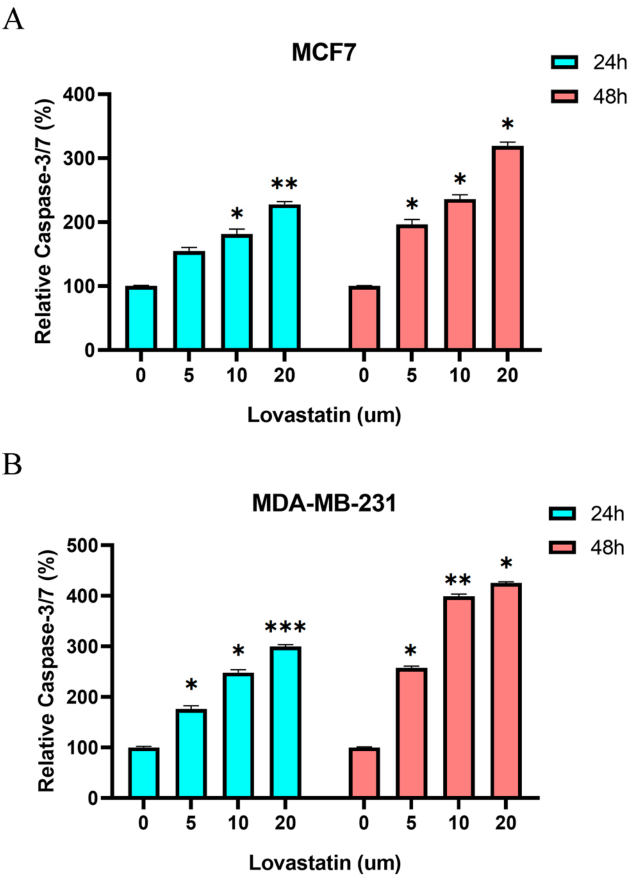

Supplementary Figure 3. Effect of lovastatin treatment at different concentrations on caspase-3/7 activity in MCF-7 (A) and MDA-MB-231 (B) cells. *, $p < 0.05$; **, $p < 0.01$; and ***, $p < 0.001$.
