# Supplementary material for: Statins inhibit paclitaxel-induced PD-L1 expression and increase CD8+ T cytotoxicity for better prognosis in breast cancer
Source: Int J Surg. 2024 May 13;110(8):4716–26. doi: 10.1097/JS9.0000000000001582 (PMC11325938; doi:10.1097/JS9.0000000000001582)
Supplement: Supplementary file 11 [file js9-110-4716-s011.pdf]

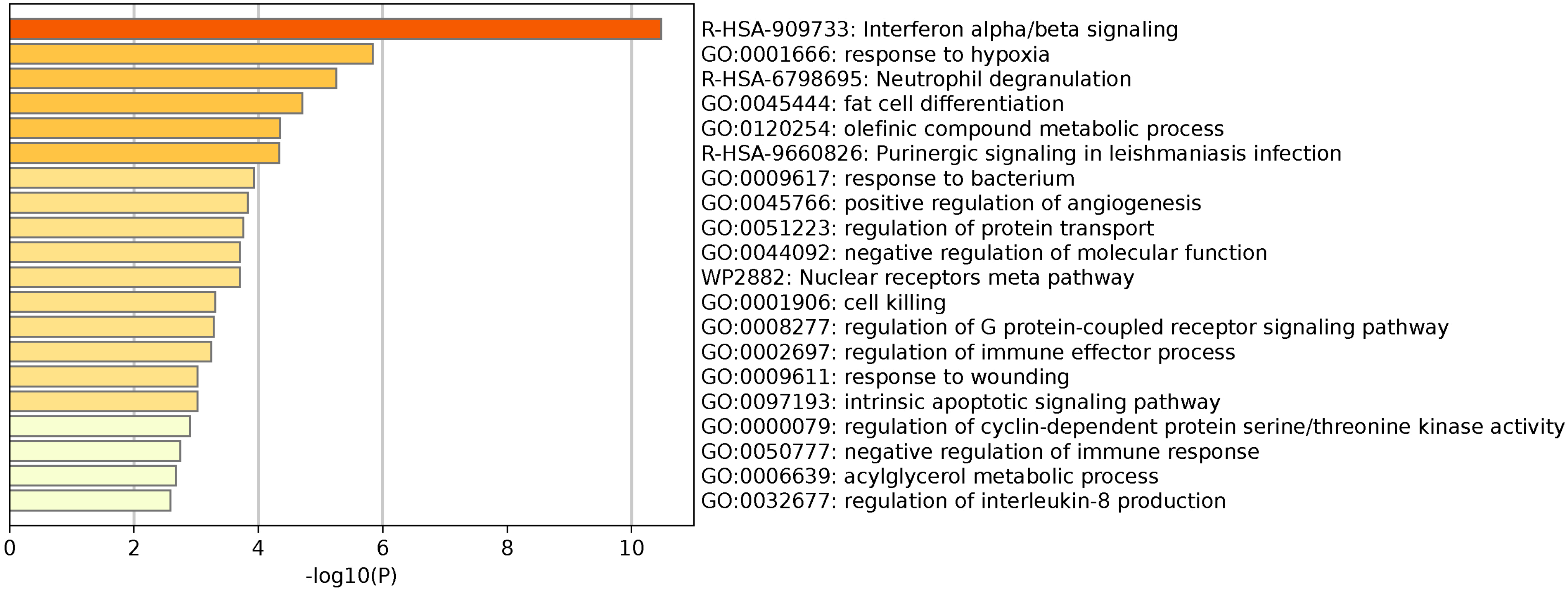

Supplementary Figure 4. Functional enrichment analysis based on metascap (https://metascape.org/gp/index.html#/main/step1).
